# Infected nonunion of tibia and femur treated by bone transport

**DOI:** 10.1186/s13018-015-0189-5

**Published:** 2015-04-10

**Authors:** Peng Yin, Lihai Zhang, Tongtong Li, Licheng Zhang, Guoqi Wang, Jiantao Li, Jianheng Liu, Jianfeng Zhou, Qun Zhang, Peifu Tang

**Affiliations:** Department of Orthopaedics, Chinese PLA General Hospital, No. 28 Fuxin Road, Beijing, 100853 P.R. China; Medical College, Nankai University, No. 94 Weijin Road, Tianjin, 300071 P.R. China

**Keywords:** Bone transport, Tibia, Femur, Infected nonunion, Systematic review

## Abstract

**Objective:**

The objective of this study was to evaluate the effectiveness of the treatment of infected nonunion of tibia and femur by bone transport.

**Material and methods:**

We retrospectively reviewed 110 patients with infected nonunion of tibia and femur treated by bone transport. Our study included 92 males and 18 females with a mean age of 38.90 years. The site of infected nonunion involved 72 tibias and 38 femurs. The mean length of the bone defects after radical debridement was 6.15 cm (range 3–13 cm).

**Results:**

The mean follow-up after removal of the apparatus was 23.12 months (14–46 months). Ten patients including seven patients with infected tibia nonunion and three patients with infected femur nonunion were lost to follow-up. All the patients achieved bone union, and no recurrence of infection was observed. The time of bone transport took a mean of 67.50 days (range 33 to 137 days), and the mean external fixation index was 1.48 months/cm (range 1.15–1.71 months/cm). According to Association for the Study and Application of the Method of Ilizarov (ASAMI) classification, bone results were excellent in 68, good in 28, fair in 12, and poor in 2; functional results were excellent in 37, good in 42, fair in 21, and no poor.

**Conclusions:**

Our study and the current evidence suggested that Ilizarov methods in the treatment of infected nonunion of tibia and femur acquired satisfied results. Radical debridement is the key step to control bone infection.

## Introduction

Infected nonunion of tibia and femur are common in clinical practice. Some coexisting problems usually complicate the nonunion including persistent infection, bone and soft tissue loss, limb-length inequalities, deformity, and joint stiffness [1,2]. Heretofore, there has still been a challenge for orthopedic surgeons about the treatment of infected nonunion of tibia and femur [3-5]. Several different surgical treatment options have been proposed, including bone grafting [6], free tissue transfer [7], antibiotic cement [4], and Ilizarov methods [8]. There are some limitations in bone grafting, such as the size of bone defects, donor site morbidity, and extended graft incorporation time [9]. Although free tissue transfer is suitable for the treatment of large bone and soft tissue loss, it is a technically demanding surgery, and it is usually associated with stress fractures and nonunion [10]. Antibiotic cement is used to control the infection efficiently, but it is only suitable for the treatment of infected nonunion with small defects or none, and bone grafting is usually necessary to achieve bone union [4]. Ilizarov methods can overcome all these difficulties and address coexisting problems simultaneously. Progressive bone histogenesis following corticotomy and bone transport help in filling bone gaps eradicating infection and promoting fracture union [2]. Therefore, bone transport has gradually been a main treatment for infected nonunion.

In the following report, we presented our experience in the treatment of infected nonunion of tibia and femur by bone transport using Ilizarov external fixator and monolateral external fixator, respectively. To our best knowledge, our study represented the largest retrospective series on the number of patients with infected nonunion of tibia and femur, and we also conducted a systematic review of infected nonunion of tibia and femur treated by Ilizarov methods.

## Patients and methods

From January 2004 to January 2013, 120 patients with infected nonunion of tibia and femur were treated in our institution. Our eligible criteria were the following: (1) patients of age of 18 years or older; (2) patients with infected tibia nonunion treated by bone transport using Ilizarov external fixator or patients with infected femur nonunion treated by bone transport using monolateral external fixator; (3) patients without an associated permanent nerve injury of the ipsilateral lower extremity; (4) no amputation. A total of 110 patients were included in our study. The study was approved by the Ethics Committee of Chinese PLA General Hospital, and written informed consent was obtained from the patients for the publication of individual clinical details and accompanying images. The study was conducted according to the ethical principles stated in the Declaration of Helsinki.

There were 92 males and 18 females with a mean age of 38.90 years (18–62 years) in our study. The site of infected nonunion involved 72 tibias and 38 femurs. The mechanisms of initial cause of injury included traffic accidents (78 patients), hitting by weight (21 patients), low energy (6 patients), high falls (3 patients), and machines (2 patients). The initial treatment of the fracture included internal fixation (60 patients), monolateral external fixation (39 patients), and hybrid external fixation (11 patients). The mean interval from the initial treatment to administration to our hospital was 25.53 months (range 4–110 months). Infected nonunion of tibia and femur existed at the time of operation in our hospital, and the mean number of previous operations was 2.61 (range 1–8 operations). The mean length of bone defects after radical debridement was 6.15 cm (range 3–13 cm), which was measured in the operation. The infection was active with purulent drainage in 80 patients, and the rest was quiescent without drainage. There were 99 complete bacterial outcomes of the samples that were obtained from purulent drainage or deep bone at the site of infected nonunion, and bacterial species grown in culture are shown in Table 1; 85 patients had bone infection with one bacterium, and 14 patients had more than one.

**Table 1:** **Proportion of bacterial species growth in culture**

**Species**	**Percent of culture**
*Staphylococcus aureus*	51%
*Pseudomonas aeruginosa*	18%
*Escherichia coli*	12%
*Klebsiella*	7%
*Enterococcus*	5%
*Acinetobacter*	2%
*Serratia*	2%
*Proteus*	1%
*Burkholderia cepacia*	1%
*Candida*	1%

Our criteria for bone grafting were that the docking line was distinct (appearance of the radiolucency at the level of the faced osteotomy surfaces) through the radiographs at the third month after the end of bone transport. Our criteria for pin-track infection diagnosis were that the clinical appearance of the pin-track was red or producing discharge or soft-tissue pain and tenderness, or both, or all of them.

### Surgical technique

The patients were positioned supine on a radiolucent table. The operative incisions were performed in accordance with previous surgical incisions when possible. Then the infected scarred soft tissue and necrotic bone were debrided radically. Cortical bone bleeding, described as the so-called paprika sign, was accepted as an indication of vital tissue [11]. Representative tissue cultures were obtained from infected tissue for the sake of finding out the infectious bacterium to choose sensitive antibiotics. If the patients had infected tibia nonunion, the Ilizarov external fixator was fixed to the tibia shaft as the way that Ilizarov rings were placed parallelly on the distal and proximal fragments and the fixed pins were inserted into the same plane under the image intensifier control; the rings in the distal or proximal fragments were fixed by three 2.5-mm pins, and the ring for bone transport in the middle was fixed by two 2.5-mm pins. The range of angle between two pins was 30°–90° in the axial plane, and we should make the angle bigger if possible. If the patients had infected femur nonunion, the monolateral external fixator was fixed to the femur shaft as the way that two or three 6-mm hydroxyapatite coated pins were inserted about 2–3 cm above and below the pre-selected osteotomy site under image intensifier control. The pins were fixed through a pre-drilled way. A 1–2-cm incision was made in order to expose the pre-selected osteotomy site, and then a subperiosteally transverse osteotomy was performed. The periosteum was sutured, and the incisions were closed with drainage tubes. If the infected site had large soft tissue defects, open dressing changing or vacuum sealing drainage (VSD) was made to close the wound.

### Post-operative protocol

All patients received a course of sensitive antibiotics for 2 to 4 weeks in intravenous way and were encouraged to perform isometric muscle and joint range-of-motion exercises on the second day after operation. The latency period before bone transport was 7–10 days, and the rate of distraction was 0.25 mm per 6 h. When bone transport was completed, the tibia or femur docked ends were compressed by 0.25 mm per day in order to provide full contact until the patient felt pain at the docking site.

Radiographs were reviewed every 2 weeks during the distraction period and monthly during the consolidation period. The Ilizarov or monolateral external fixator was removed when radiographs showed solid docking-site union and the regenerate area had a minimum of three complete cortices. Bone results and functional results were evaluated according to Association for the Study and Application of the Method of Ilizarov (ASAMI) classification [12-14].

## Results

**Figure 1:**
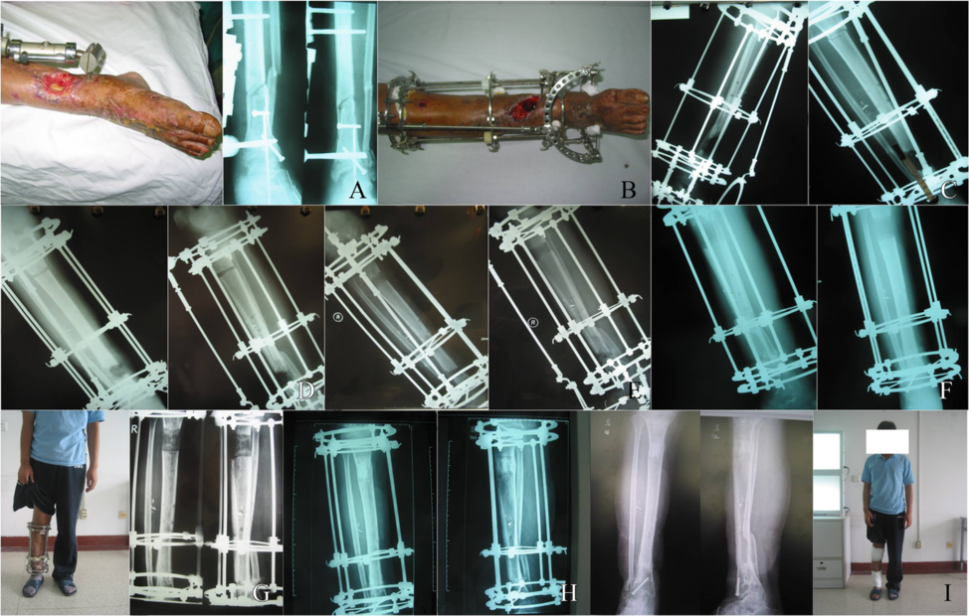
**A 19**-**year-old man who had infected tibia nonunion. (A)** This is a 19-year-old man who had infected tibia nonunion with soft tissue defected and foot drop. **(B)** It shows the picture in operation. **(C)** Debridement of the site of infected nonunion with 4-cm bone defects and corticotomy of tibia. **(D)** Two weeks after operation with bone transport. **(E)** Four weeks after operation with bone transport. **(F)** Six weeks after operation, bone ends contacted with each other at the docking site and the regenerated bone had begun to be mineralized. **(G)** Three months after operation, foot drop was corrected and the frame of foot was removed. **(H)** Five months after operation, good consolidation of the regenerate and complete bone union at the docking site was presented. **(I)** Seven months after operation, it shows complete bone union, and the apparatus was removed.

**Figure 2:**
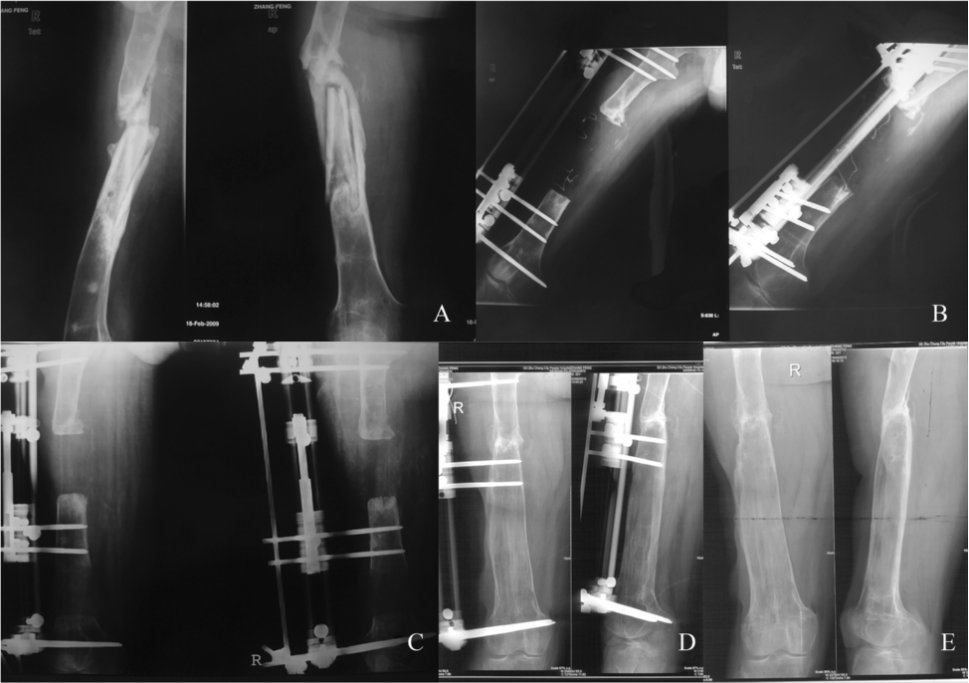
**A 33-year-old man who had an infected femur nonunion. (A)** Radiograph of a 33-year-old man who had an infected femur nonunion. **(B)** Debridement of the site of infected nonunion with 8-cm bone defects and corticotomy of femur. **(C)** Two months after operation with bone transport. **(D)** Twelve months after operation, good consolidation of the regenerate and complete bone union at the docking site was presented. **(E)** The external fixator was removed at 12 months after operation.

The mean follow-up after removal of the apparatus was 23.12 months (range 14–46 months). Ten patients including seven patients with infected tibia nonunion and three patients with infected femur nonunion were lost to follow-up, because they moved and changed telephone number and could not be contacted. We were unable to evaluate the final functional outcomes in these ten patients. All the patients achieved bone union. The time of bone transport took a mean of 67.50 days (range 33 to 137 days) in all patients, and a mean of 69.11 days (range 33 to 137 days) in the patients with infected tibia nonunion, and a mean of 64.45 days (range 45–86 days) in the patients with infected femur nonunion. The mean external fixation index was 1.48 months/cm (range 1.15–1.71 months/cm) in all patients, and 1.48 months/cm (range 1.15–1.67 months/cm) in the patients with infected tibia nonunion, and 1.50 months/cm (range 1.28–1.71 months/cm) in the patients with infected femur nonunion (Figures 1 and 2).

According to ASAMI classification, bone results and functional results were evaluated, and the details are listed in Tables 2 and 3.

**Table 2:** **Evaluation of the bone results**

**Bone results**	**Infected tibia nonunion**	**Infected femur nonunion**	**Total**	**Criteria**
Excellent	46	22	68	Union, no infection, deformity <7°, limb length discrepancy (LLD) <2.5 cm
Good	17	11	28	Union plus any two of the following: absence of infection, deformity <7°, LLD <2.5 cm.
Fair	7	5	12	Union plus any one of the following: absence of infection, deformity <7°, LLD <2.5 cm.
Poor	2	0	2	Nonunion/refracture/union plus infection plus deformity >7° plus LLD >2.5 cm

**Table 3:** **Evaluation of the functional results**

**Functional results**	**Infected tibia nonunion**	**Infected femur nonunion**	**Total**	**Criteria**
Excellent	25	12	37	Active, no limp, minimum stiffness (loss of <15° knee extension/<15° ankle dorsiflexion), no reflex sympathetic dystrophy (RSD), insignificant pain.
Good	27	15	42	Active, with one or two of the following: limb, stiffness, RSD, significant pain
Fair	13	8	21	Active, with three or all of the following: limb, stiffness, RSD, significant pain
Poor	0	0	0	Inactive (unemployment or inability to return to daily activities because of injury)
Failure	0	0	0	Amputation

### Complications

All the patients had a feeling of pain during the distraction period, but they could have tolerated the pain by oral analgesics. Pin-track infection occurred in 70 patients which involved 46 infected tibia nonunion and 24 infected femur nonunion. Sixty-six patients had only local inflammation, and they were treated by pin care and empirical broad spectrum antibiotics for oral administration; four patients had a purulent drainage, and aspiration of pus were cultured for selecting sensitive antibiotics, and finally they were treated by intravenous sensitive antibiotics. Thirty-two patients had the axial deviation during bone transport including 20 infected tibias nonunion and 12 infected femur nonunion, and external fixator adjustments for modification were performed in these patients. Bone grafting was required in 12 patients which involved 7 infected tibia nonunion and 5 infected femur nonunion at the docking site. Loosening of wires or pins occurred in 8 patients involving 4 infected tibia nonunion and 4 infected femur nonunion. The loose wires were re-tensioned, and the loose pins which are due to loss of adhesion with bone were removed and the new pins were inserted. Two patients with infected tibia nonunion suffered refracture at the docking site after removal of the external fixator, and then were re-applied with the Ilizarov external fixators, and finally achieved bone union. There were no neurovascular complications or a compartment syndrome.

## Discussion

This is a retrospective study of bone transport in the treatment of the largest number of patients with infected nonunion of tibia and femur at present. The present study showed that infected nonunion of tibia and femur treated by bone transport acquired satisfied results. The excellent and good rate of bone results was 87.27% (96/110), and the excellent and good rate of functional results was 79% (79/100). All the patients achieved bone union and no recurrence of infection was observed.

In order to further demonstrate the effectiveness of Ilizarov methods and give a better guideline for clinical treatment of infected nonunion, we conducted a systematic review of infected nonunion of tibia and femur treated by Ilizarov methods. We searched literatures from the PubMed, Cochrane Library, EMBASE, and other relevant English orthopedic journals between January 1995 and April 2013. The initial literature search identified 337 relevant records, and finally 22 studies and a total of 426 patients were included in the systematic review [2,13,15-34]. Mean age, mean bone defects, bone union, bone results, functional results, complications per patient, external fixation time, external fixation index, and amputation rate were recorded and statistically analyzed using weighted means based on the sample size in each study by SPSS 13.0. The following data were calculated: The mean age was 33.774 ± 4.665 years [2,11-30]; the mean bone defects was 6.527 ± 1.882 cm [2,13,15-22,24-34]; the bone union rate was 96.935% [2,11-30]; the mean complications per patient was 1.567 ± 0.901 [2,13,15-22,24-27,29-34]; the mean external fixation time was 10.845 ± 5.339 months [2,13,15-18,21-34]; the mean external fixation index was 1.579 ± 0.585 months/cm [2,13,15,16,21,22,24-34]; the good and excellent rate in bone results was 87.38% (range 45.5%–100%) [2,13,15,19,20,25-27,29-34]; the good and excellent rate in functional results was 74.18% (range 27.8%–97.1%) [2,13,15,16,19,20,22,25-27,29-33]; the average of poor rate in functional results was 7.89% (range 0%–20%) [2,13,15,16,19,20,22,25-27,29-33]; and the average of amputation rate was 4.94% (3.57%–10%) [15,17,20,25]. More details are listed in Table 4.

**Table 4:** **The treatment of infected nonunion of tibia and femur by Ilizarov methods**

**Author**	**Technique**	**PN**	**MA (years)**	**MBD (cm)**	**Site**	**Bone union (%)**	**Bone results (excellent/good/fair/poor)**	**Functional results (excellent/good/fair/poor)**	**Complications (per patient)**	**EFT (months)**	**EFI (M/cm)**
Feng	RD, AT, BT (IEF)	21	34.6	6.6	Tibia	21/21(100%)	19/2/0/0(Paley)	__	0.4(8/12)	9.8	1.48
Arora	RD, AT, BT (MEF)	15	29	7.9	Femur	15/15(100%)	12/3/0/0(Arora)	5/8/2/0(Arora)	1.33(20/15)	7.3	0.93
Liu	RD, AT, BT (MEF), 5 F	35	37.3	3.5	Tibia	35/35(100%)	28/5/2/0(Paley)	30/4/1/0(Paley)	1.11(39/35)	10.7	1.36
Sala	RD, AT, BT (IEF/TSF)	12	44	8	Tibia	12/12(100%)	10/2/0/0(ASAMI)	6/5/1/0(ASAMI)	2.08(25/12)	13.9	2.0
Blum	RD, AT, BT (IEF)	50	29.9	8.8	Femur	49/50(98%)	__	__	__	24.5	2.8
Megas	RD, AT, CO, or ACL (IEF)	9	39.7	5	Tibia	9/9(100%)	5/4/0/0(Paley)	3/4/2/0(Paley)	1.44(13/9)	7.83	1.07
Bumbasirevic	RD, AT, BT (IEF)	30	30.4	6.9	Tibia	29/30(97%)	19/10/0/1(Paley)	13/14/2/1(Paley)	1.4(42/30)	9.7	1.48
Emara	RD, AT, BT (IEF), BG RD, AT, BT (IEF and IMN), BG	33	29	6	Tibia	16/16(100%)	15/1/0/0(ASAMI)	12/1/3/0(ASAMI)	0.4(6/16)	8.5 3.1	1.5
					Tibia	17/17(100%)	17/0/0/0(ASAMI)	13/2/2/0(ASAMI)	0.12(2/17)		0.55
Madhusudhan	RD, AT, ACL (IEF) RD, AT, BT (IEF)	22	37.2	4/5.4^a^	Tibia	13/13(100%)	4/3/4/2(ASAMI)	1/3/6/2(ASAMI)*	2.73 (60/22)	9.3 8.5	2.33
					Tibia	9/9(100%)	0/3/4/2(ASAMI)	0/1/3/2(ASAMI)**			1.57
Magadum	RD, ACL (IEF)	27	39	10	Tibia	24/25(96%)***	19/5/0/1(ASAMI)	15/8/1/1(ASAMI)	1.16(29/25)	10.2	1.02
Krishnan	RD, AT, BT, or ACL (IEF)	20	38.4	6	Femur	19/20(95%)	13/4/1/1(ASAMI) One amputation	3/9/3/4(ASAMI) One amputation	3.55(71/20)	7.8	1.28
Sadiris	RD, AT, ACL, or BT (IEF)	13	34.6	8.3	Femur	13/13(100%)	8/4/1/0(Paley)	3/4/4/2(Paley)	0.76(10/13)	10.33	1.24
Abdel-Aal	RD, BT (IEF)	9	30.66	10.7	Tibia	9/9(100%)	__	__	1.22(11/9)	12.78	1.22
MaHale	RD, AT, BT, or ACL or CO (IEF)	10	31	__	Tibia	10/10(100%)	__	__	__	9.0	__
Arora	RD, BT, or CO (IEF)	46	35	6	Tibia/Femur	44/46(95.4%)	__	15/16/13/2(Paley)	0.74(34/46)	8.7	1.33
Atesalp	RD, AT, BT (IEF), 3 F	14	25	4.4	Tibia	13/14(92.9%)	__	__	1.21(17/14)	6.8	1.55
Barbarossa	RD, AT, BT (IEF)	23	40.7	6.2	Tibia	20/23(87%)	8/8/2/4(ASAMI) One amputation	2/10/6/4(ASAMI) One amputation	3.35(77/23)	__	__
Maini	RD, AT, BT (IEF)	15	27.4	7	Tibia/Femur	15/15(100%)	7/3/0/5(ASAMI)	4/7/1/3(ASAMI)	2.06(31/15)	__	__
Laursen	RD, AT, BT, or CO (IEF)	9	25.78	4.89	Tibia	9/9(100%)	__	__	1.56(14/9)	6.7	__
Ring	RD, BT, or ACL or CO (IEF),3 F	10	34	4.3	Tibia	9/10(90%)	__	__	1.8(18/10)	6.9	__
Hosny	RD, BT, or CO (IEF), 3AT	11	27	3.7	Tibia	11/11(100%)	__	5/3/2/1(Cattaneo)	1.27(14/11)	8.5	2.3
Dendrinos	RD, BT (IEF)	28	37	6	Tibia	25/28(89%)	14/8/1/5(ASAMI)	7/11/4/5(ASAMI) One amputation	2.5(70/28)	10	1.67

*ACL* acute compression and lengthening, *ASAMI* Association for the Study of the Method of Ilizarov, *AT* antibiotics treatment, *BG* bone graft, *BT* bone transport, *CO* compression osteosynthesis, *EFI* external fixation index, *EFT* external fixation time, *F* flaps, *IEF* Ilizarov external fixator, *IMN* intramedullary nailing, *MA* mean age, *MBD* mean bone defects, *MFU* mean follow-up, *MEF* monolateral external fixator, *PN* patient number, *RD* radical debridement, *TSF* Taylor Spatial Frame.

*1 patient lost for follow-up.

**3 patients were unable to evaluate.

***2 patients lost for follow-up.

^a^The study included two groups, the mean bone defects is 4 cm in one group, and 5.4 cm in another group.

__ The data were not reported in the studies.

In our study, the complications per patient were 1.13% (124/110), which was less than the average data recorded in the aforementioned systematic review. We believed that making full preparations for the surgery based on specific conditions of every patient, meticulous post-operative care, and patients’ good compliance could decrease the complication rates. The most common complication was pin-track infection, and the incidence was 63.64% (70/110). Although we paid more attention to pin-track care, there was still a high incidence. Therefore, in addition to pin-track care, we recognized that pin-track infection was also related to patient’s bone quality, immunity, and so on. The rate of axial deviation during bone transport was 29.09% (32/110). The complication could be corrected by external fixator adjustments. We considered that the reason was overload of weight bearing or excessive functional exercise, and it may be avoided by reasonable rehabilitation exercises. There is a controversy on regular bone grafting at the docking site. We did not perform regular bone grafting, and bone grafting was required in only 12 patients (10.91%, 12/110) in our study. The rates of loosening of pins and refracture were 7.27% (8/110) and 1.82% (2/110), respectively, which were relatively lower. Good and excellent rate in bone results was 87.27%, and good and excellent rate in functional results was 79% in our study, these data was similar to the average data recorded in the aforementioned systematic review.

In our experience, the infected union of femur is more suitable for bone transport by monolateral external fixator due to the extensive soft-tissue envelope around the femur and the neurovascular structures. Monolateral external fixators for bone transport are simple in design and application and easy to carry out during a surgical procedure while maintaining it, and it can also alleviate pain caused by pin during the distraction period compared to the Ilizarov external fixator. The infected tibia is more suitable for bone transport by the Ilizarov external fixator, because Ilizarov external fixators for bone transport can provide a stable mechanical environment, correct deformities, and enable weight bearing during the course of treatment, and the patients can also tolerate the pain caused by tensioned wires of Ilizarov external fixators. In addition, some important aspects of bone transport should be paid attention. (1) We should perform radical debridement in the site of infected nonunion. This is the key step to control bone infection. (2) Distraction usually begins between 7 and 10 days after the operation at a rate of 0.25 mm per 6 h. If regenerate quality is poor, the speed of distraction will slow down.

In conclusion, our study and the current evidence suggested that Ilizarov methods in the treatment of infected nonunion of tibia and femur acquired satisfied effects in bone results and functional results. Radical debridement is the key step to control bone infection. However, our study lacks a direct comparison with any other treatment options, further randomized controlled trials are needed to draw more valuable conclusion.
